# Tissue slide-based microRNA characterization of tumors: how detailed could diagnosis become for cancer medicine?

**DOI:** 10.1586/14737159.2014.944507

**Published:** 2014-08-04

**Authors:** Lorenzo F Sempere

**Affiliations:** ^a^ Laboratory of microRNA Diagnostics and Therapeutics, Van Andel Research Institute, 333 Bostwick Ave, N.E, Grand Rapids, MI 49503, USA

**Keywords:** biomarker, contextual information, diagnostics, formalin-fixed paraffin-embedded tissues, *in situ* hybridization, locked nucleic acid, miRNA, miR, multiplex assay

## Abstract

miRNAs are short, non-coding, regulatory RNAs that exert cell type-dependent, context-dependent, transcriptome-wide gene expression control under physiological and pathological conditions. Tissue slide-based assays provide qualitative (tumor compartment) and semi-quantitative (expression levels) information about altered miRNA expression at single-cell resolution in clinical tumor specimens. Reviewed here are key technological advances in the last 5 years that have led to implementation of fully automated, robust and reproducible tissue slide-based assays for *in situ* miRNA detection on US FDA-approved instruments; recent tissue slide-based discovery studies that suggest potential clinical applications of specific miRNAs in cancer medicine are highlighted; and the challenges in bringing tissue slide-based miRNA assays into the clinic are discussed, including clinical validation, biomarker performance, biomarker space and integration with other biomarkers.

MicroRNAs (miRNAs) are an evolutionarily conserved class of short, non-coding, regulatory RNAs that generally control gene expression at the post-transcriptional level [1–5]. A single miRNA can interact with hundreds of target messenger RNAs (mRNAs) and affect the protein output of a large number of target genes at once [6–8]. In some instances, specific miRNAs can coordinately regulate the expression of multiple protein components of the same signaling pathway or biological process. Thus, deregulation of miRNA activity can have broad and deleterious consequences for cell homeostasis and can contribute to human disease [9–14]. Over the last decade, rapid technological advances in platforms for high-throughput miRNA profiling in tissue and blood samples have generated lists and signatures of miRNAs associated with various cancer types, neurodegenerative conditions and cardiovascular disease [9,10,15,16]. Several quantitative real-time PCR-based assays have been commercialized as laboratory-developed tests (LDTs) for use in tissue samples to differentially diagnose pancreatic ductal adenocarcinoma, to diagnose the subtypes of kidney and lung cancer, and to identify the organ site of cancers of unknown primary origin [17–23]; a handful of these tests are already being reimbursed for by health insurance companies [24,25]. Some of the disease-associated miRNAs have also been functionally characterized in preclinical models and some front-runner compounds are already being evaluated in clinical trials for anti-viral and oncology applications [26,27].

Recently, more attention has been paid to the cell types in which miRNA expression is altered in tumor tissues. Tumors are complex quasi-organs that contain cancer cells and several other supportive and reactive cell types in the tumor microenvironment. Intra- and inter-tumoral heterogeneity at the molecular and cellular levels [28–37] can confound and mislead the interpretation of miRNA expression analyses that use RNA extracted from tumor tissue or blood [15,16,38,39]. Over the last 5 years, various groups have developed robust and reproducible *in situ* hybridization (ISH) methods to visualize miRNA expression within individual cells in formalin-fixed paraffin-embedded clinical tissue specimens, because of the clinical implications of the cell type (tumor compartment) that harbors altered miRNA expression.

This review focuses on the implementation of tissue slide-based assays for miRNA ISH detection in cancer and the implications for cancer medicine. It provides a brief historical perspective of the origins of these tissue slide-based assays and describes technological advances that rapidly led from manual single-marker assays to fully automated multiplex assays for the co-detection of miRNA and protein markers. These technological advances are intimately intertwined with the potential clinical applications of miRNA biomarkers. These tissue slide-based assays provide powerful investigational tools to better understand the etiological relevance of miRNAs and, eventually, could provide diagnostic tools to guide treatment decisions. The review highlights recent tissue slide-based discovery studies that assess the diagnostic and prognostic value of miRNAs in common cancer types including breast, colon, lung, pancreas and skin. The final topic deals with considerations and challenges to further implement lead miRNA indicators into useful biomarkers that deliver solutions for unmet clinical needs in cancer medicine.

## High-throughput expression profiling of miRNAs in cancer tissues

The first link between aberrant miRNA expression and function in human cancer was found in 2002, when chromosomal deletion of the *mir-15/mir-16* locus and/or altered expression of miR-15/miR-16 were associated with chronic lymphocytic leukemia [40]. Subsequently, miR-15/miR-16 were functionally linked to disease etiology via regulation of a key target gene, *BCL2* [41]. Soon after, various groups developed different high-throughput detection platforms, including bead-based hybridization and microarray chips, to profile the expression of known miRNAs in panels of different hematologic and solid cancer types [42–51]. Over the years, improvements in these platforms as well as newer technologies such as multiplex RT-PCR plates, RNA-sequencing and Nanostring have enabled an in-depth characterization of microRNome changes in many cancer types [52–55].

### Validation of miRNA expression profiling signatures

Technical validation of differentially expressed miRNAs identified via high-throughput platforms typically consists of single miRNA detection by a stem-loop RT-PCR assay [56] or a similar approach. While there are technical discrepancies and different biases of each detection method [54,55], miRNA expression signatures have been technically validated in multiple studies. However, none of these detection methods enable us to identify cellular source(s) of altered miRNA expression. Most studies have used RNA extracted from whole tumor tissues or normal reference controls. It has often been assumed that altered miRNA expression in whole-tumor tissue biopsies or in blood reflects molecular changes within cancer cells. Accordingly, functional studies have been carried out in cancer cell lines to understand the molecular mechanism(s) behind deregulated miRNA expression, which may lead to new therapeutic opportunities [13,14]. A predominant deregulation of miRNA activity in cell types other than cancer cells would put into question the interpretation of functional assays with cancer cell lines and would force us to reconsider the etiological contribution of particular miRNAs to carcinogenesis. Thus, the confirmation of altered miRNA expression within individual cells in the native context of a complex tumor lesion is required to unequivocally assign miRNA dysregulation to cancer cells or other cells of the tumor microenvironment.

Some studies have enriched tissue samples for specific cellular content (cancer cells vs stroma) using laser capture microdissetion [57–60], a tissue core punch extractor [61] or tissue macrodissection [62] before miRNA detection with high-throughput and sensitive methods. However, these approaches for enrichment are labor-intensive and technically demanding, but still do not enable miRNA detection within individual cells. ISH is an ideal assay for validating miRNA expression at single-cell resolution using a limited amount of a clinical tissue specimen. Moreover, miRNA detection by ISH follows a procedure very similar to tissue slide-based morphology-driven clinical assays for protein detection by immunohistochemistry (IHC) and DNA detection by fluorescence ISH. The principles and clinical applications of miRNA detection by ISH are presented in following sections.

## Tissue slide-base assays for in situ miRNA detection

*In situ* detection of miRNA expression was first achieved in vertebrate embryos using whole-mount ISH protocols [63,64]. The key innovation was the incorporation of locked nucleic acid (LNA)-modified antisense probes. LNAs are high-affinity RNA analogs that have unprecedentedly strong and specific binding properties [65,66] to complementary short RNA sequences such as those of mature miRNAs. Whole-mount ISH results confirmed expression profiling findings of mammalian organ- and cell-type-specific miRNA expression patterns [67,68]. Soon after that key innovation, various groups implemented chromogenic and fluorescent-based ISH assays using LNA-modified probes to detect miRNA expression in frozen and fresh clinical tumor specimens to validate expression profiling signatures [51,69–74]. Nelson *et al*. [51] used single digoxigenin-tagged LNA-modified probes against miRNAs (miR-9, miR-124, miR-125b) differentially expressed in brain cancer; the probes were detected using anti-digoxigenin antibody conjugated to alkaline phosphatase and chromogenic development of the 4-nitroblue tetrazolium and 5-bromo-4-chloro-3-indolyl phosphate substrate. Sempere *et al*. [69] used single fluorescein (fluorescein isothiocyanate [FITC])-tagged LNA-modified probes against miRNAs (let-7a, miR-21, miR-145, miR-125b miR-205, miR-451) in breast cancer; the probes were detected with anti-FITC antibody conjugated to horseradish peroxidase (HRP) and deposition of reactive tyramide substrate conjugated to FITC. Both studies demonstrated that altered miRNA expression in tumors was complex and involved distinct normal cell types and tumor compartments for different miRNAs; some expression changes suggested an etiological contribution of specific miRNAs, whereas others did not. These early studies set the basis for a workable assay.

### Methodological implementation & protocol variations

Over the last 5 years, several groups have considerably improved these chromogenic and fluorescent-based ISH assays for miRNA detection in formalin-fixed paraffin-embedded tissue specimens (Table 1). The limit of detection of the ISH assay is low compared with exponential amplification methods such as quantitative real-time PCR; thus, detection of miRNAs expressed at low copy number is technically difficult. The author describes below general strategies and variants that have been implemented for optimal *in situ* detection of miRNA expression (Table 1). The author highlights some critical parameters; more thorough technical details and general troubleshooting tips have been presented elsewhere [75–79].

**Table 1.** **Variations and evolution of tissue slide-based assays for *in situ* detection of miRNAs^†^.**

**miRNA probe**	**Tag**	**Label**	**Multiplex**	**Tissue**	**Procedure**	**Ref.**
miR-9, miR-124, miR-125b	DIG	Chromo^NBT/BCIP^	N/D	Brain	Manual	[51]
miR-21	FITC	Chromo^DAB^	N/D	Colon	Manual	[71]
miR-150	DIG	Chromo^NBT/BCIP^	N/D	Colon, Lymphoid	Manual	[106,118]
miR-21, miR-155	DIG	Chromo^NBT/BCIP^	N/D	Pancreas	Manual	[72]
Let-7b, miR-205	DIG	Fluorescent		Breast^TMA^	Manual	[103]
let-7a, let-7b, let-7c, let-7f, let-7g, miR-98	DIG	Chromo^DAB^	N/D	Breast^TMA^	Manual	[107]
let-7a, miR-21, miR-125b, miR-145, miR-205, miR-451	FITC	Fluorescent	N/D	Breast^TMA^, Lung^TMA^	Manual	[69,142,143]
miR-21, miR-126, miR-155, miR-182, miR-210	DIG2X	Chromo^NBT/BCIP^	N/D	Lung^TMA^	Manual	[82–86]
miR-146, miR-375	DIG2X	Fluorescent or Chromo^NBT/BCIP^	N/D	Lung, Esophagus	Manual; 2-fluoro RNA probes	[87,88]
miR-200a	DIG2X	Chromo^NBT/BCIP^	N/D	Breast^TMA^	Manual	[89]
miR-21	DIG2X	Chromo^NBT/BCIP^	N/D	Pancreas^TMA^	Manual	[90]
Let-7a, miR-1, miR-21, miR-103, miR-124, miR-126, miR-143, miR-145, miR-205, miR-223	DIG2X	Chromo^NBT/BCIP^	N/D	Brain, Breast, Colon	Manual	[77,78,91]
miR-205	DIG2X	Fluorescent	ISH/IFplex: 1 RNA + 1 protein	Prostate	Manual	[92]
miR-124	FITC	Fluorescent	ISH/IFplex: 1 RNA + 1 protein	Brain	Manual; HIER instead of PK	[99]
let-7a, let-7b, let-7c, let-7d, let-7e, let-7f, let-7glet-7i, miR-1, miR-7a, miR-9, miR-9*, miR-15a, miR-16, miR-21, miR-22, miR-26a, miR-26b, miR-27a, miR-28, miR-29a, miR-30a, miR-30d, miR-99a, miR-99b, miR-101a, miR-122, miR-124, miR-126*, miR-128a, miR-128b, miR-129, miR-130a, miR-133a, miR-132, miR-134, miR-135b, miR-140, miR-143, miR-153, miR-191, miR-370, miR-410, miR-434, miR-5p, miR-467a, miR-497, miR-744, miR-872	DIG	Fluorescent	IHC/ISHplex: 1 protein + 1 RNA	Mouse tissues	Manual; Post-fixation with EDC	[79]
miR-205, miR-375	FITC6X, Bio6X	Fluorescent	ISHplex: 3 RNAs	Skin	Manual; Post-fixation with EDC	[81]
miR-21, miR-34a, miR-92a, miR-205, miR-221	DIG	Fluorescent	ISH/IHCplex: 1 RNA + 1 protein	Breast^TMA^ Skin^TMA^	Manual	[101,102]
miR-142–5p	DIG	Fluorescent	ISH/IFplex: 1 RNA + 2 proteins	Brain	Manual; EDC fixation post-HIER	[100]
miR-1, miR-125b, miR-199a	DIG2X	Fluorescent	ISH/IFplex: 1 RNA + 2 proteins	Heart	Manual	[93]
miR-21, miR-205	DIG2X	Fluorescent	ISH/IHCplex: 1 RNA + 1 protein	Breast, Colon	Semi-automated	[94]
let-7a, miR-10b, miR-21, miR-24, mir-34a, miR-34c-5p, miR-125b, miR-126, miR-141, miR-143, miR-145, miR-155, miR-196, miR-200b, miR-205, miR-214, miR-221, miR-375, miR-451	FITC2X, Bio2X, DIG2X, BrdU2X	Fluorescent	ISH/IHCplex: 4 RNAs + 1 protein 2 RNAs + 3 proteins 1 RNA + 4 proteins	Breast, Pancreas^FNA^, Colon, Lung, Prostate	Semi-automated	[75,95,96]
miR-21	DIG2X	Chromo^NBT/BCIP^	N/D	Colon	Fully- automated	[97,98]
miR-21, miR-126, miR-145	DIG2X	Chromo^NBT/BCIP^	ISH/IHC2plex: 1 RNA + 1 protein	Mouse and rat tissues	Fully- automated	[80]
let-7a, miR-10b, miR-21, miR-34a, miR-126, miR-145, miR-155, miR-205, miR-210	FITC2X	Fluorescent	ISH/IHC4plex: 2 RNAs + 2 proteins 1 RNAs + 3 proteins	Breast	Fully- automated	[76]

^†^This table summarizes main technical features and capabilities of tissue-slide based assays for *in situ* detection of miRNAs in formalin-fixed paraffin-embedded normal and tumor tissues from various organ sites. Unless otherwise indicated, *in situ* miRNA detection was performed on whole-tissue sections. From the top to the bottom of the list, the evolution of this assay can be appreciated by the implementation of innovative features and technological advances, from multi-tag probes to fully automated performance. Bio: Biotin; BrdU: Bromodeoxyuridine; Chromo^NBT/BCIP^ and Chromo^DAB^: A chromogenic stain with alkaline phosphatase-mediated development of 4-nitroblue tetrazolium and 5-bromo-4-chloro-30-indolyl phosphate substrate or horseradish peroxidase-mediated development 3,3’-diaminobenzidine substrate, respectively; DIG: Digoxigenin; FITC: Fluorescein; FNA: Fine-needle aspirate; IHC: Immunohistochemistry (enzyme conjugated to antibody develops signal); ISH: *In situ* hybridization; IF: Immunofluorescence (signal directly conjugated to antibody); TMA: Tissue microarray.

#### Preanalytical sample preparation

- Tissue fixation in formalin for less than 24 h or more than 72 h before for paraffin embedding and other sample preparation steps such as time from operating room to tissue processing can affect miRNA detection [38,80]. These can be confounding factors when analyzing miRNA expression from tissue specimens collected in different institutions and at different times, especially in historical archives dating back to the 1980s and 90s, before standardization of tissue processing procedures. A post-fixation step with 1-ethyl-3-(3-dimethylaminopropyl) carbodiimide (EDC) [79,81] can be also used to increase the retention of miRNA molecules. However, EDC fixation may limit the multiplex capability of miRNA and protein co-detection because EDC cross-links can cause epitope masking [79].

#### Probe design

- LNA-modified nucleotides are typically incorporated every 2–3 nucleotides to increase the melting temperature (Tm) by 10–15°C. Ideally, the Tm for a probe should be about 75°C, but Tm prediction formulae are not always accurate. Empirical determination of Tm for optimal probe detection includes variables such as probe concentration, ionic strength and formamide concentration in the hybridization solution, time and temperature of hybridization and temperature and stringency of saline sodium citrate washes. Reticular nuclear and/or nucleolar stains typically indicate spurious hybridization of the probe against ribosomal or other abundant RNAs. Thus, it is important to have adequate control tissues and/or reference cell lines to assess the specificity of probe binding.

#### Probe tags

- The addition of multiple hapten tags – most commonly two terminal tags (e.g., DIG2X, FITC2X) [75–78,82–98], but at times up to six (FITC6X, Bio2X) [81] – increases probe detection.

#### Probe access to fixed miRNA

- Enzymatic pretreatment with proteinase K is commonly used to digest tissue and break down protein–RNA crosslinks to facilitate probe penetration and miRNA exposure. Changes in time, temperature and concentration of proteinase K between samples (intra-experimental) and between experiments (inter-experimental) affect stains by failing to sufficiently open up the tissue or by causing too much tissue damage [38,78]. Heat-induced retrieval with citrate buffer can be used [99,100] instead of proteinase K. This pretreatment may be beneficial for co-detection of miRNAs and proteins if concerns exist that proteinase K may destroy protein epitopes. However, various groups have reported multiplex co-detection of miRNAs and a score of proteins after proteinase K pretreatment [75,76,80,92–96].

#### Detection methods

- Chromogenic or fluorescent-based stains have been used to detect miRNA probes. Chromogenic staining can be achieved by alkaline phosphatase-mediated development of 4-nitroblue tetrazolium and 5-bromo-4-chloro-3-indolyl phosphate substrate and/or HRP-mediated development of 3,3′-diaminobenzidine substrate. Fluorescent staining can be achieved by HRP-mediated deposition of reactive substrates conjugated to a variety of fluorochromes (FITC, rhodamine, Dyglight dye series, Alexa dye series, Cy dye series). The use of antibody sandwich amplification (e.g., anti-tag primary antibody followed by secondary antibody-conjugated to HRP) [38,75,76] and/or enzymatic polymers (e.g., poly-HRP) [76,80] increases the number of functional units (e.g., HRP units) per bound probe molecule and thereby increases label deposition. The use of fluorescent stains (enzyme-reactive substrates conjugated to fluorochromes) can increase signal sensitivity and dynamic range of detection and can also enable a variety of multiplexing applications for high-content marker analysis [16,81,93–95,101].

Several research groups have already implemented these assays in fully automated staining stations [76,80,97,98], some of which are US FDA-approved for diagnostic IHC assays. Automation enables precise and consistent performance of time-sensitive steps such as proteinase K pretreatment and substrate incubation for signal development. These robust and reproducible ISH assays now meet technical standards of, use same instrumentation as and are compatible with diagnostic IHC assays.

### High-content image analysis

The scoring of miRNA expression takes into account the tumor compartment and the intensity of the stain. Some studies rely on visual examination by independent viewers, who report intensity of staining on a 0–3 scale and score cases as low or high, noting the tumor compartment (cancer vs stroma). Some studies have used matched normals as a reference for intensity changes in the tumor. This reporting system is very similar to what is used for protein stains in the clinic. As with protein stains, it is assumed that tissues are handled, processed and preserved under identical conditions, so that any difference in stain intensity reflects expression changes. This may not be always the case. Some studies have used expression of small nuclear RNA U6 or ribosomal RNA 18S as a control for RNA quality and integrity [38,95,101,102]. However, the expression of these control RNA markers varies depending of disease stage. A universal RNA control for miRNA normalization in tissue slide-based assays or other detection methods has not yet been established. Notwithstanding this limitation, various groups have demonstrated that ISH-based stainings are reproducible and semi-quantitative [95,97,101,103]. Direct comparison on the same tissue samples indicate that ISH assays can discriminate a similar range of fold changes in expression as RT-PCR assays [97,101,103].

To further standardize and objectify staining scores, several groups have begun to employ computer-assisted image analysis tools [38,76,95,97,98,101–105]. For example, fluorescent staining of let-7a and miR-205 in the cancer cell compartment of breast cancer tissue core sections was analyzed with an Ariol automated microscopic image capture system [103]. Chromogenic staining of miR-21 in the stromal compartment of colon cancer whole-tissue sections was analyzed with a Visiopharm integrated microscope and software module; morphological characteristics indicated that miR-21 was predominantly expressed in tumor-associated fibroblasts [97]. Chromogenic staining of miR-126 in blood vessels of lung cancer whole-tissue sections was also analyzed with the Visiopharm system [104]. In addition to morphological characteristics, co-stains with cell type-specific proteins in multiplex assays can more unambiguously identify individual or a small nest of cancer cells (e.g., cytokeratin markers for carcinomas), tumor-associated fibroblasts (e.g., smooth muscle actin), endothelial cells (e.g., CD31) and leukocyte lineages and subpopulations (e.g., CD3 for T-cell lymphocytes, CD11b for myeloid cells, CD68 for macrophages) [38,76]. Fluorescent staining of miR-221 in the cancer cell compartment of breast cancer tissue core sections was analyzed with Automated Quantitative Analysis technology; co-staining with pan-cytokeratin marker was used to define cancer cell compartment [101]. Fluorescent staining of miR-21 in the stromal compartment of whole-tissue sections of breast and colon cancer cases was analyzed with Image-Pro Plus; co-staining with vimentin and smooth muscle actin markers confirmed that miR-21 was predominantly expressed in tumor-associated fibroblasts [38].

## Discovery studies in cancer using tissue slide-based assays for in situ miRNA detection

Recent tissue slide-based discovery studies suggest potential clinical applications of specific miRNAs in variety of cancers, including breast, colon, lung, pancreas and skin (Table 2). Most of the studied miRNAs had been identified as cancer-associated by high-throughput expression profiling analyses. These tissue slide-based studies for the first time evaluated the effect of the tumor compartment on the diagnostic and prognostic value of altered miRNA expression. In one such study, the prognostic value of miR-150 was assessed in colon cancer by RT-PCR and ISH assays [106]. Although expression analysis was determined in two independent patient cohorts, it is worth noting that the ISH-based miR-150 score was more powerful in stratifying patients who had better clinical outcomes [106]. The author highlights below the main findings of some other tissue-slide studies that reinforce the importance of contextual miRNA expression analysis.

**Table 2.** **Potential clinical applications of miRNA-based *in situ* hybridization assays^†^.**

**Cancer**	**Patient cohort**	**miRNA**	**Expressing cells**	**Informative cells**	**Clinical application**	**Ref.**
Bladder	229 urothelial carcinoma cases: stage 0–1 (79%) versus stage II–IV (21%)	miR-34a	Epithelial/tumor cells^TMA^	Tumor cells	Positive indicator for recurrence-free survival in stage 0–I cases [HR: 0.57; p = 0.04]	[Andrew AS, Cowper R, Marsit CJ, *et al.* Expression of tumor suppressive miRNA-34a is associated with a reduced risk of bladder cancer recurrence, Submitted]
Brain	193 glioma cases: grade I–III (19%) versus grade IV (81%, glioblastomas)	miR-21	Tumor cells, tumor-associated vessels	Tumor cells	Negative prognostic indicator [HR: 1.545; p = 0.049]	[105]
Breast	2033 population-based breast carcinoma cases: ER+ (84%) versus ER– (16%)	let-7b	Epithelial/tumor cells^TMA^	Tumor cells	Positive prognostic indicator in ER+ and/or PR+ cases [HR: 0.79; p = 0.02]	[103]
	80 breast carcinoma cases: ER+ (44%) versus ER– (56%); stage I/II (56%) versus stage III/IV (44%)	let-7g	Luminal epithelial/tumor cells^TMA^	Tumor cells	Positive prognostic indicator [p = 0.021; univariate]	[107]
	901 non-metastatic breast carcinoma cases: ER+ (76%) versus ER–(24%); stage I(68%) versus stage II (32%)	miR-21	Tumor cells, tumor-associated fibroblasts^TMA^	Tumor cells or tumor-associated fibroblasts depending on molecular subtype	Negative prognostic indicator [HR: 1.96; p < 0.001]	[Mackenzie TA, Schwartz G, Calderone H, *et al*. Stromal expression of miR-21 identifies high-risk group in triple negative breast cancer, Submitted]
	102 breast carcinoma cases: ER+ (49.6%) versus ER– (65.7%)	miR-27	Tumor cells^TMA^	Tumor cells	Negative prognostic indicator [HR: 3.57; p = 0.025]	[108]
	1686 population-based breast carcinoma cases: ER+ (81%) versus ER– (19%)	miR-205	Myoepithelial/tumor cells^TMA^	Tumor cells	Positive prognostic indicator in ductal histology cases [HR: 0.77; p = 0.02]	[103]
	20 ER–, PR–, HER2– breast carcinoma cases		Myoepithelial/tumor cells^TMA^	Tumor cells	Positive prognostic indicator in ER–, PR–, HER2– cases [trend only]	[69]
	377 breast carcinoma cases	miR-221	Tumor cells	Tumor cells	Positive prognostic indicator [HR: 0.70; p = 0.032]	[101]
Colon	129 stage II colon cancer cases	miR-21	Tumor cells, tumor-associated fibroblasts	Tumor-associated fibroblasts	Negative prognostic indicator [HR: 1.28; p = 0.012]	[97]
	764 stage II colon cases		Tumor cells, tumor-associated fibroblasts	Tumor-associated fibroblasts	Negative prognostic indicator [HR: 1.92; p = 0.008]	[98]
	89 metastatic colorectal cancer cases treated with first-line capecitabine and oxaliplatin (XELOX)	miR-126	Endothelial cells in primary tumor tissues	Endothelial cells	Positive predictive indicator of response to XELOX treatment [p < 0.0001]	[104]
					Positive prognostic indicator	[104]
	185 colorectal cases	miR-150	Epithelial/Tumor cells	Tumor cells	Positive prognostic indicator [HR: 0.18; p < 0.01]	[106]
					Positive predictive indicator of response to adjuvant chemotherapy	[106]
Esophagus	249 esophageal squamous cell carcinoma cases with paired N/T	miR-375	Esophageal cells/Tumor cells^TMA^	Tumor cells	Positive prognostic indicator [p = 0.04, univariate]	[88]
Lung	335 NSCLC cases: stage I (47%), stage II (40%), stage III (13%); node negative (69%) versus node positive (31%)	miR-21	Tumor cells, tumor-associated fibroblasts^TMA^	Tumor cells	Positive prognostic indicator in node positive cases [HR: 0.49; p = 0.027]	[85]
			Tumor cells	Tumor-associated fibroblasts	Negative prognostic indicator [p = 0.002, univariate]	[85]
	312 NSCLC cases: stage I (47%), stage II (40%), stage III (13%)	miR-126	Tumor cells and others	Tumor cells	Negative prognostic indicator [HR: 1.78; p = 0.01]	[83]
	43 NSCLC cases	miR-146	Tumor cells	Tumor cells	Negative prognostic indicator [HR: 10.56; p = 0.003]	[87]
	320 NSCLC cases: adenocarcinoma (34%, AC) versus SCC (66%) carcinomas; node negative (69%) versus node positive (21%)	miR-155	Tumor cells^TMA^	Tumor cells	Negative prognostic indicator in AC cases [HR: 1.87; p = 0.047]	[82]
					Positive prognostic indicator in node positive SCC cases [HR: 0.45; p = 0.039]	[82]
	305 NSCLC cases: stage I (47%), stage II (42%), stage III (9%); adeno- (37%), squamous cell (62.6%, SCC), large cell (0.4%) carcinomas	miR-182	Tumor cells and stromal cells^TMA^	Tumor cells	Positive prognostic indicator in stage II cases [HR: 0.5; p = 0.02]	[84]
					Positive prognostic indicator in SCC cases [HR: 0.57; p = 0.048]	[84]
	259 NSCLC cases: stage I (47%), stage II (40%), stage III (13%)	miR-210	Tumor cells and stromal cells^TMA^	Stromal cells	Positive prognostic indicator [HR: 1.9; p = 0.013]	[86]
Lymphoid	36 lymphoid cases: 28 BL versus 8 reactive lymphoid hyperplasia	miR-150	Lymphoid/Tumor cells	Tumor cells	Differential diagnostic marker for BL [p = 0.001]	[118]
Pancreas	106 cases: benign (10%) versus PDAC (90%)	miR-10b	Tumor cells, others cell types^FNA^	Tumor cells	Differential diagnostic marker for PDAC [p = 0.001]	[95]
	95 PDAC cases: stage I/II (52%) versus stage III/IV (48%)		Tumor cells, others cell types^FNA^	Tumor cells	Negative prognostic indicator [HR: 3.1; p = 0.032]	[95]
	145 PDAC cases: stage I (37%) versus stage II (62%)	miR-21	Tumor cells, tumor-associated fibroblasts^TMA^	Tumor-associated fibroblasts	Negative prognostic indicator [HR: 1.4; p = 0.004]	[90]
	80 PDAC cases		Tumor cells^TMA^	Tumor cells	Negative prognostic indicator in node negative cases [p = 0.037, univariate]	[70]
Prostate	169 prostatic cancer cases	miR-21	Tumor cells	Tumors	Negative indicator for biochemical recurrence [p < 0.001, univariate]	[144]
Skin	297 melanoma cases: 105 training set + 192 validation set	miR-205	Tumor cells^TMA^	Tumor cells	Positive prognostic indicator [HR: 0.511; p = 0.048 – validation set]	[102]
	16 cases: four BCC versus 12 MCC	miR-205	Tumor cells	Tumor cells	Differential diagnostic marker for BCC	[81]
		miR-375	Tumor cells	Tumor cells	Differential diagnostic marker for MCC	[81]

^†^This table summarizes clinical characteristics of patient cohorts in which altered miRNA expression was determined by tissue slide-based assays. Statistically significant associations between contextual information of the tumor compartment and the levels of miRNA expression and clinical parameters are reported. Positive prognostic indicators denote that high levels of the miRNAs are positively correlated with good outcome (HR < 1) and, conversely, negative prognostic indicators indicate that high levels of the miRNAs are negatively correlated with good outcome (HR > 1). Unless otherwise indicated, HR for outcome refers to cancer-specific or overall survival in all comers as determined by multivariate COX regression analyses. Unless otherwise indicated, *in situ* miRNA detection was performed on whole-tissue sections. BCC: Basal cell carcinoma; BL: Burkitt lymphoma; HR: Hazard ratio; FNA: Fine-needle aspirate; MCC: Merkel cell carcinoma; NSCLC: Non-small-cell lung cancer; PDAC: Pancreatic ductal adenocarcinoma; SCC: Squamous cell carcinoma; TMA: Tissue microarray.

### Altered miRNA expression in cancer cells

High let-7g and miR-221 expression and low expression of miR-27 in the cancer cell compartment of breast cancer cases are associated with better clinical outcome [101,107,108]. miR-205 is expressed at high levels in myoepithelial and basal epithelial cells in normal breast and skin tissue, respectively; retention of miR-205 expression in cancer cells is associated with better clinical outcomes in both breast cancer and melanoma cases [69,102,103]. High miR-182 expression and low miR-126 and miR-146 expression in the cancer cell compartment are associated with better outcomes in lung cancer cases [83,84,87]. The cancer cell expression of miR-126 is intriguing, because miR-126 is a well-characterized miRNA for its role in endothelial cell biology and has been shown to have expression confined to endothelial cells [38,109–115]. Future studies are needed to validate the prognostic value of miR-126 expression in lung cancer cells.

### Altered miRNA expression in stromal cells

miR-21 is expressed in cancer cells and stromal cells (mainly tumor-associated fibroblasts) in several solid cancers [38,69–71,90,91,97,98,116]. In colon and pancreas cancer, most cases predominantly express miR-21 in the stroma, and this is the only tumor compartment with prognostic value [90,97,98]. *In vitro* co-culture studies of cancer cells and tumor-associated fibroblasts isolated from colon and pancreatic cancer patients have revealed fibrogenic roles of miR-21, suggesting a functional etiological contribution of miR-21 to support cancer cell growth and enhance malignancy [90,117]. In lung cancer, it appears that high expression of miR-21 in stromal cells is a negative prognostic indicator, whereas high expression in the cancer cells of node-positive cases is protective [85]. In contrast, high expression of miR-21 in cancer cells and tumor-associated blood vessels in glioma cases are associated with poor clinical outcome [105]. The difference between expression patterns of miR-21 in carcinomas and gliomas may reflect the different idiosyncrasies of cancers of epithelial and neural origin, respectively. In an interesting study, high levels of miR-126 in endothelial cells in primary tumor tissues were associated with treatment response to first-line capecitabine and oxaliplatin chemotherapy and with better clinical outcome in advanced colorectal cancer cases with metastatic disease [104]. On a separate study, expression of hypoxia-induced miR-210 was detected in cancer and stromal cells in lung cancer cases, but only high levels of miR-210 in stromal cells were significantly associated with a better outcome [118].

### Study design limitations

**Figure 1.**
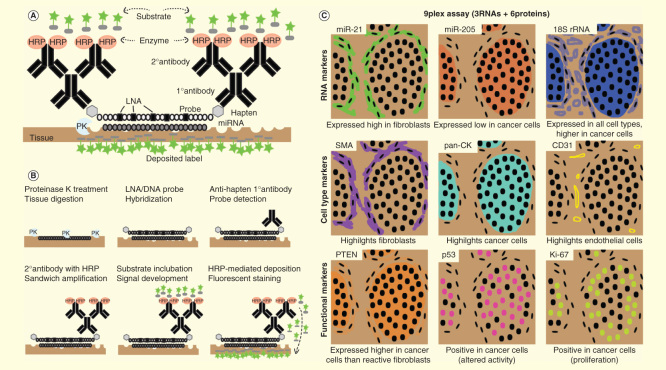
**Automated platform for high-content marker analysis of tumor tissues. (A)** Key components of *in situ* miRNA detection assay. **(B)** Purposes of key components and the steps they take part in, from tissue digestion to covalently linked deposition of fluorescent labels. **(C)** Virtual staining rendition of a 9plex assay that co-detects three RNA markers (two miRNAs + control rRNA) and six protein markers (three cell type-specific + three functional/prognostic markers); black dots indicate nuclei revealed by counterstaining with DAPI. Top left, representative miR-21 stain (green) on an ER^−^PR^−^HER2^−^ breast cancer case in the first round of HRP-mediated deposition of reactive substrate conjugated with a fluorochrome. Sequential rounds of HRP-mediated deposition of substrates conjugated with spectrally unique fluorochromes are carried out to detect probes against other RNA markers with appropriate anti-hapten primary and secondary antibody combinations or protein markers with appropriate primary and secondary antibody combinations. Each new staining round requires a peroxide block step to inactive HRP from the preceding round.

These tissue slide-based discovery studies reported statistically significant prognostic indications for compartment-specific altered miRNA expression in multivariate analyses that adjusted for standard clinicopathologic prognostic factors (Table 2). These studies suggest that prognostic information obtained with miRNA expression is independent of standard prognostic factors. However, the sample size and/or limited molecular characterization of tumor specimens precluded a more in-depth evaluation of specific miRNAs in the context of known molecular biomarkers that impact clinical decisions (e.g., estrogen receptor [ER]/progesterone receptor [PR]/human epidermal growth factor-like receptor 2 [HER2] status in breast cancer; K-Ras and EGFR mutation status in colon and lung cancer). Nonetheless, a handful of adequately powered studies with larger case sets (n > 500) have started to find links to specific molecular alterations. In breast cancer, the status of the ER, PR and HER2 are used for diagnostic, prognostic and treatment decisions. Within major subtypes defined by the combined status of ER/PR/HER2, patients still exhibit heterogeneous responses to treatment and clinical outcome [119]. Low expression of tumor suppressive let-7b in cancer cells from ER+ and/or PR+ (with or without HER2+) cases was associated with a better clinical outcome [103]. High expression of oncogenic miR-21 in cancer cells from ER+ and/or PR+ HER2– cases and in tumor-associated fibroblasts from ER–, PR–, HER2– cases was associated with a poorer clinical outcome [Mackenzie TA, Schwartz G, Calderone H, et al. Stromal Expression Of miR-21 Identifies High-Risk Group In Triple Negative Breast Cancer, Submitted]. Although more studies and clinical validation of let-7b and miR-21 indications are necessary, these early works suggest ways in which miRNA biomarkers could complement current clinically used protein markers to improve prognostic assessment and to guide treatment decisions (Figure 1).

HRP: Horseradish peroxidase.

## Expert commentary

Cancer cell clonal diversity, tissue heterogeneity and the tumor microenvironment influence the efficacy of current treatments of solid tumors. The detailed cellular and molecular characterization of tumors presents a unique opportunity to translate scientific knowledge into actionable clinical information. Over the last 5 years, several groups have overcome technical challenges of detecting miRNA using ISH assays in fixed tumor tissues. These tissue slide-based assays enable the characterization of miRNA expression in individual cells in the tumor microenvironment and the determination of which tumor compartment(s) harbors this altered expression. This type of assay enhances the precision and accuracy of informative miRNA biomarkers for which context and morphological features are important. However, the use of tissue slide-based miRNAs in clinical application is still in an early stage of development relative to RT-PCR-based assays, which have already entered the clinic as LDTs. Thus, it is important to consider the advantages and limitations of each platform from the technical, instrumental and diagnostic vantage points [15,16], so as to better focus efforts on the development of tissue slide-based assays that can uniquely shed light on knowledge gaps and address unmet clinical needs in cancer medicine.

- Tissue analysis using tissue slide-based assays provides a direct evaluation of disease state based on miRNA expression (or other biomarkers) for diagnostic and prognostic purposes, but it is not generally an appropriate biological material for early disease detection or disease monitoring. For such applications, RT-PCR-based miRNA detection in blood (serum, plasma, cellular component, exosome fraction) or other biological fluids, which can be collected by non-invasive means at frequent intervals, is being actively pursued by many research laboratory and diagnostic companies [120].
- Tissue slide-based assays can be more cost–effective in screening the expression of selected miRNAs (n < 10) in a large number of tissue samples (n > 500) using tissue microarrays, whereas RT-PCR assays provide high throughput in terms of the number of independent miRNAs (n > 100) that can be detected in a single tube reaction.
- Tissue slide-based assays can more precisely and more readily provide contextual information for abundantly expressed miRNAs, whereas RT-PCR assays are more sensitive and can be used to detect all known miRNAs.
- Tissue slide-based assays enable co-detection of miRNAs and other bioanalytes (DNA, mRNA, lncRNAs, rRNAs, snRNAs, tRNAs) in their native tissue, cellular and subcellular context on the same tissue section. Integrative approaches and multi-marker contextual signatures could considerably enhance the diagnostic power of individual components.
- Tissue slide-based assays enable co-detection of miRNAs and their cognate target mRNAs or target gene products (protein). These assays can be used to validate miRNA and target gene interactions at the mRNA (multiplex ISH) or protein (combined ISH/IHCplex) levels and to assess whether miRNA regulatory networks are engaged and functional in individual tumor cases.

For some specific questions and applications, tissue slide-based assays could outperform RT-PCR assays. This point was illustrated in previous section by the cancer cell expression of miR-150 [106], the endothelial cell expression of miR-126 in colon cancer [104] and the stromal cell expression of miR-21 in breast, colon and pancreatic cancer [90,91,97] [Mackenzie TA, Schwartz G, Calderone H, et al. Stromal Expression Of miR-21 Identifies High-Risk Group In Triple Negative Breast Cancer, Submitted]. Future tissue slide-based discovery and validation studies may identify a clinical value of miRNAs that did not pass quality or statistical filters in previous expression profiling studies using mixed RNA pools from whole tissue samples. In addition, there may be viable business development opportunities to build on and eventually replace RT-PCR assays. The Asuragen miRInform® PANCREAS is a multiplex RT-PCR assay that measure levels of two miRNAs to improve the detection and diagnosis of pancreatic ductal adenocarcinoma [24]. This and other LDTs, including the Rosetta lung cancer test [25], use fewer than 10 miRNAs to establish informative test results. Thus, these tests would be amenable to adaptation for tissue slide-based assays.

For other clinical applications, in which a miRNA signature of 10 or more is required to establish informative test results, RT-PCR-based assays are a more appropriate platform. The Rosetta cancer origin test is an example of such a multiplex RT-PCR-based assay, which measures expression of a large miRNA signature (64 miRNAs) to identify likely primary tumor types using primary or metastatic tumors [25]. This test may be particularly helpful for cancers having an unknown primary, as correct diagnosis would allow treatment using the standard of care for that particular cancer type. Currently, IHC assays with a battery of cell type-specific markers are needed to identify or confirm origin (after the Rosetta cancer origin test). ISH detection of informative miRNAs by the Rosetta cancer origin test could be used to analytically confirm test results before or in conjunction with IHC markers. Together, these RT-PCR-based LDTs offer successful examples that could guide a business development plan for tissue slide-based miRNA diagnostics and inform regulatory procedures required by FDA and other regulatory agencies.

### Clinical implications of tissue slide-based assays

Besides the potential of tissue slide-based assays to be more informative than RT-PCR assays, a question with broader implications is whether miRNAs can be more informative, advantageous or versatile than other biomarkers for tissue analysis. RT-PCR-based detection of miR-205 for differential diagnosis of non-small-cell lung cancer adenocarcinomas versus squamous cell carcinomas is a case in point [121–125]. Would ISH miR-205 detection provide any additional value to the current multi-protein panels (e.g., TTF-1, napsin A, p63, cytokeratin 5/6) [126,127] in making this determination? While it seems unlikely that miR-205 could displace such well-established panels, miR-205 may also have prognostic value in squamous cell carcinomas [128]; thus, a combined ISH/IHC of this miRNA and protein could expand current application of individual markers. In general, it would be important to consider the availability and performance of known biomarkers to identify areas in which miRNAs can be particularly useful. For example, it would seem impractical to use miRNA expression to obtain information similar to the well-established ER/PR/HER2 status in breast cancer, but miRNA expression could be of use in cancer cells or other tumor compartments to refine the characterization and prognostic indications based on ER/PR/HER2 status. When useful IHC markers are not available, developing a miRNA biomarker program could have unique advantages. Since specific probes and other reagents to detect miRNA expression can be readily synthesized based on Watson–Crick sequence complementarity rules, tissue slide-based assays of lead miRNA biomarkers could be expeditiously implemented for disease assessment, such as cancer cell-expression of miR-34 for recurrence risk stratification in bladder cancer [Andrew AS, Cowper R, Marsit CJ, et al. Expression of Tumor Suppressive microRNA-34a Is Associated With A Reduced Risk Of Bladder Cancer Recurrence, Submitted].

## Five-year view

Automated pipelines exist for whole-slide image acquisition and computer-assisted image analysis [129], as mentioned above. Some of these platforms integrate multispectral analysis capabilities which could enable detection of as many as 10–12 markers in a single tissue slide using spectrally distinct fluorochromes. As the field of digital pathology and associated quantitative image analysis tools mature [129,130], it is foreseeable that these technologies could be applied for high-content analysis of miRNA expression in translational pathology research laboratories as well as in clinical laboratories. High-content multi-panel assays would contain miRNAs, reference RNAs and cell type-specific and prognostic protein markers to maximize contextual information per tissue slide for diagnostic and prognostic applications. A contextual signature of 20–36 miRNA and protein markers (containing up to 9 miRNAs) could be generated using 2–3 consecutive tissue sections, which would be in a similar range of marker numbers as commercially available prognostic gene expression signatures (e.g., Oncotype Dx, 21 mRNAs; PAM50/Prosigna, 50 mRNAs) [131]. The ancillary protein markers would refine cell identity and the complementary prognostic markers would dissect the molecular status of miRNA-expressing cells in cancer cells, reactive stromal elements such as tumor-associated fibroblasts and immune cells such as T cells and macrophages (Figure 1). Whole-slide image analysis would also enable a deeper understanding of tumor tissue composition and heterogeneity, namely, how local and regional distribution in response to hypoxia, inflammation and pH changes may affect cells at the molecular level and in cell–cell interactions. Thus, it could be technologically feasible to integrate ISH/IHC multiplex assays for high-content morphology-driven analysis of informative miRNA and protein biomarkers in clinical settings (Figure 1).

While miRNA biomarkers continue to generate great interest and hold great potential, they need to be subject to same scrutiny and achieve the same benchmark performance as other bioanalytes for clinical implementation [132–134]. So far altered miRNA expression has an exploratory biomarker status. For most tissue slide-based *in situ* miRNA detection, analytical and clinical validation is still required. A hurdle for clinical validation is to have access to high-quality tissue specimens from large cohorts of patients with well-annotated clinicopathologic features and outcome data. National programs exist, such as the Cancer Therapy Evaluation Program [135] from the National Cancer Institute in the USA, which provide valuable biospecimen resources for retrospective and prospective miRNA biomarker studies. A large number of tissue specimens could be analyzed, first in screening tissue microarrays to cover the entire patient cohorts in an expedited and economic study and then in follow-up analysis with individual whole tissue sections for miRNAs that warrant further evaluation as diagnostic, prognostic or predictive biomarkers.

Purely prognostic miRNA biomarkers may inform tumor biology and provide lead candidates for therapeutic intervention in the translational cancer research setting. However, their immediate impact on clinical decisions may be limited as they would not inform specific course of treatment, other than perhaps suggest a more aggressive intervention plan. Predictive miRNA biomarkers that indicate a specific treatment approach or selection may be more desirable as it may have a more direct impact on clinical decisions. Further development as predictive or integrated biomarkers for specific treatment recommendation in tissue specimens collected during multi-arm clinical trials evaluating different treatment combinations is now possible thanks to Cancer Therapy Evaluation Program and similar national programs. For example, tissue specimens from several breast cancer clinical trials are available for researchers to evaluate the predictive value of compartment-specific miR-21 expression in specific subtype breast cancer cases treated with different combination of hormonal and/or chemotherapy. In this context, the predictive value of miRNA would not likely reflect a direct effect of the drug treatment on miRNA activity as these drugs do not target the miRNA. In contrast, expression analysis of a miRNA that is the intended target of a drug could serve as an integral predictive biomarker in miRNA-based therapy trials. miR-34-based restoration therapy using a synthetic compound that mimics pre-miR-34 is in a Phase I clinical trial for cancer patients with liver involvement, either primary or metastatic tumors [26]. Restoration therapies of tumor suppressive let-7 [136,137] and immunomodulatory miR-155 activities [138] as well as inhibition therapies of tumor promoting and fibrogenic miR-21 activities [90,117,139,140] have been recently proposed and may be clinically evaluated in the near future [141]. Tissue slide-based determination of expression levels of targeted miRNA, which closely correlate with miRNA activity, would provide personalized and precise selection of patients who would more likely benefit from miRNA-based therapeutic interventions.

In conclusion, the technology and resources are now available to generate high-content contextual signatures that integrate miRNA biomarker information, but is the biology relevant and robust enough to make a clinical impact? Clinical validation of lead miRNA biomarkers will be the first step toward clinical implementation and commercialization of tissue slide-based miRNA detection assays.

## Notes

Key issues

- The use of altered miRNAs as new biomarkers for diagnostic, prognostic and therapeutic applications in cancer medicine is a rapidly growing field. Various detection methods exist to measure changes of miRNA levels.
- Tissue slide-based assays for *in situ* miRNA detection on tumor tissues offer unique advantages over other methods of determining miRNA expression in individual cells (tumor compartment) and of registering the location and distribution (intratumoral heterogeneity) of the miRNA-expressing cells.
- Tissue slide-based assays characterize miRNA expression in its intact and native tumor context, in contrast to free miRNA in solution that is used for quantitative real-time PCR or other detection methods.
- Tissue slide-based assays can provide more precise and accurate diagnostic information because they provide qualitative and semiquantitative information of altered miRNA expression at single-cell resolution.
- Recent tissue slide-based discovery studies suggest the diagnostic and prognostic potential of altered miRNAs in specific tumor compartments. Cancer cells are not the only tumor compartment in which altered miRNA expression can be informative. Indeed, altered expression of miR-21 in the stromal compartment (mainly tumor-associated fibroblasts) provides prognostic information in breast, colon and pancreas cancer.
- These tissue slide-based *in situ* hybridization assays are compatible and can be combined with standard clinical immunohistochemistry assays. miRNA and protein diagnostic information can be integrated in a single tissue section.
- Recent advances in multispectral analysis and digital pathology enable whole-slide high-content image analysis. Multiplexing of up to 12 markers is feasible using work flow that is comparable to the routine/standard procedures in translational pathology research and clinical laboratories.
- Clinical validation of lead miRNA biomarkers in large prospective and longitudinal studies is required to ignite the interest of clinicians to consider using miRNA in their practice and of industry partners in developing business plans for miRNA-based clinical tools.
